# Ultrasound Elastography in Inflammatory Bowel Diseases: A Systematic Review of Accuracy Compared with Histopathological Assessment

**DOI:** 10.1093/ecco-jcc/jjac082

**Published:** 2022-06-13

**Authors:** Arianna Dal Buono, Francesco Faita, Laurent Peyrin-Biroulet, Silvio Danese, Mariangela Allocca

**Affiliations:** IBD Center, Department of Gastroenterology, Humanitas Clinical and Research Center - IRCCS, Rozzano, Milan, Italy; Italian National Research Council Institute of Clinical Physiology, Pisa, Italy; Department of Gastroenterology and Inserm NGERE U1256, University Hospital of Nancy, University of Lorraine, Vandoeuvre-lès-Nancy, France; Gastroenterology and Endoscopy, IRCCS Ospedale San Raffaele, and University Vita-Salute San Raffaele, Milan, Italy; Gastroenterology and Endoscopy, IRCCS Ospedale San Raffaele, and University Vita-Salute San Raffaele, Milan, Italy

**Keywords:** Elastography, shear waves, bowel ultrasound, inflammation, inflammatory bowel disease

## Abstract

**Background and Aims:**

Ultrasound elastography [USE] is an innovative, non-invasive, promptly available, ancillary technique that has been proposed in the evaluation of intestinal fibrosis as a monitorable biomarker, in terms of stiffness. The non-invasive estimate of fibrosis by USE appears appealing for dedicated physicians, in order to optimise the treatments for inflammatory bowel disease [IBD] patients [surgical vs non-surgical]. We aimed to systematically review literature evidence on ultrasound elastography in IBD patients.

**Methods:**

For this qualitative systematic review, we searched PubMed, EMBASE, and Scopus to identify all studies, published until October 2021, investigating the application of USE in IBD patients compared with histopathological assessment.

**Results:**

Overall, 12 papers published between 2011 and 2019 were included. A total of 275 IBD patients were included: 272 Crohn’s disease [CD] [98.9%] and three ulcerative colitis [UC] [1.1%]. Seven [58.3%] and four [41.6%] studies investigated strain elastography [SE] and shear wave elastography [SWE], respectively; in one study [0.1%] both techniques were addressed. The histological evaluation was largely conducted on surgical specimens and in two studies endoscopic biopsies were also included. The histological assessment was semi-quantitative in all the included studies, except for two where the fibrosis was evaluated only qualitatively. In 10/12 publications USE could accurately distinguish inflammation from fibrosis in the examined bowel tracts.

**Conclusions:**

From the preliminary available data, an overall moderate-to-good accuracy of USE in detecting histological fibrosis [10/12 studies] was found. Point-shear wave elastography has been shown to perform superiorly. Further studies are needed to confirm these evidences.

## 1. Introduction

Chronic inflammatory bowel diseases [IBDs] are relapsing-remitting and progressive conditions that lead to irreversible bowel damage.^1,2^ Especially the stricturing phenotype of Crohn’s disease [CD] and late stages of ulcerative colitis [UC] are characterised by the development of fibrosis in the affected bowel tract.^3,4^

Fibrotic strictures have a multifactorial biological basis that involves the activation of mesenchymal cells that over-produce and deposit extracellular matrix.^3^ Soluble molecules such as cytokines and growth factors [i.e., transforming growth factors, tumour necrosis factor, interleukins] trigger this activation, with a subsequent remodelling of the tissue by matrix metalloproteinases and other fibrogenic enzymes.^4,5^

The predominancy of fibrosis is believed to be less responsive to medical treatments and often requires a surgical intervention [i.e., resection, strictureplasty].^6,7^ For this reason, distinguishing between IBD patients with a primarily inflammatory or a fibrotic disease has a relevant impact on clinical management.

In a recent systematic review, the sensitivity in detecting fibrosis of cross-sectional imaging techniques has been assessed at around 80% for both computed tomography [CT] and magnetic resonance imaging [MRI].^8^ The main features that help the radiologists in distinguishing between inflammation and fibrosis are bowel wall thickness, mural contrast enhancement, mesenteric vascularity, and mesenteric fat stranding.^9–11^

To date, neither a scoring system nor standardised criteria have been established for differentiating fibrosis at cross-sectional imaging, thus remaining an unsolved challenge for dedicated physicians.

Ultrasound elastography [USE] is an innovative, non-invasive, promptly available, ancillary technique that has been proposed in the evaluation of intestinal fibrosis as a monitorable imaging biomarker, in terms of stiffness.^8^

As concerns technical aspects, USE assesses the elastic properties of soft tissues by acoustic or mechanical stimulation: the tissue response to the stress is processed and codified as an image with a scale of colours or as a quantitative measurement corresponding to the estimated stiffness value. The main types of USE are shear wave elastography [SWE] and strain elastography [SE]. The stimulus for the measured stress ranges from acoustic radiation force impulse imaging [ARFI] to mechanical or physiological palpation. In detail, point-SWE [pSWE] estimates a quantitative value of a specific point of the examined tissue, whereas two-dimensional SWE [2D-SWE] codifies a colour map that reflects the stiffness of a wider portion of the examined tissue. The application of USE has already been incorporated in the diagnostic algorithms of diseases of the liver, breast, pancreas, and thyroid, especially for neoplastic lesions.^12,13^ Thanks to recent technological advancements, USE is implemented and usable in real time. However, there are no current international guidelines instructing on the applications of elastography in the field of IBD.

The role of USE in the management of IBD is currently under investigation, and its validation requires precise knowledge of the corresponding histological features. So far, data from literature on USE accuracy in the field of IBD are mostly derived from small cohorts and have never been comprehensively reviewed specifically and exclusively in comparison with histology as a reference standard. The purpose of our systematic review is to provide an exhaustive overview of the available data on USE in IBD patients.

## 2. Technique and principles of ultrasound elastography

**Figure 1.**
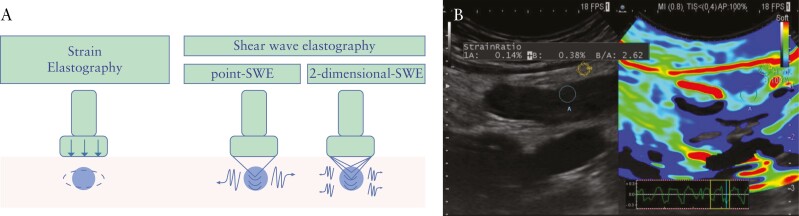
Technique and principles of ultrasound elastography. A. The technologies currently used and commercially available in ultrasound [US] machines are divided into two main types: strain [SE] and shear wave elastography [SWE]. In SE, the induced tissue displacement is traced between pairs of echo frames, then the strain is calculated from their gradient. Technically, two SWE methods can be distinguished: the point-SWE [pSWE] and the 2-dimensional-SWE [2D-SWE]. In the p-SWE, the speed of shear wave is measured in a single specific location [ROI]; the 2D-SWE produces a quantified colour map of the distribution of shear wave velocities in a wider region. B. An example of SE applied to bowel wall is shown. SWE: shear wave elastography.

Elastography evaluates the tissue elasticity, defined as the tendency of that tissue to resist deformations by an applied force, or to return to its original shape once the force is removed. Biologically, a stiff region displays less deformation compared with healthy surrounding tissue when the same stress stimulus is applied. The technologies currently used and commercially available in US machines are divided into two main types: strain [SE] and shear wave elastography [SWE] [Figure 1]. These types of elastography differ in the process used to measure tissue deformation in response to an applied force. In detail, the applied force could be a mechanical internal [exploiting physiological periodic compression induced by circulatory and/or respiratory motion] or external [generated by hand through the US transducer that is gently pressed against explored tissues] pressure [Figure 1]. Alternatively, tissues can be stressed by imposing a low-frequency ARFI stimulus generated by the US device itself. In SE, the induced tissue displacement is traced between pairs of echo frames and then the strain is calculated from their gradient. Through the use of a colour map, the different strains are encoded within a two-dimensional image that can be instantly visualised together with the conventional B-mode US image. The SE is a semi-quantitative technique that cannot measure the elasticity of the examined tissue as an absolute value, since the absolute value of the applied stress is unknown.

In SWE, the dynamic stress induces shear waves that propagate perpendicular to the US beam. The speed of the generated shear waves is measured and returns quantitative estimates of the tissue elasticity. Technically, two SWE methods can be distinguished: the point-SWE [pSWE] and the 2-dimensional-SWE [2D-SWE]. In the p-SWE, the speed of the shear wave is measured in a single specific location [ROI]; the 2D-SWE produces a quantified colour map of the distribution of shear wave velocities in a wider region.

## 3. Methods

This work was conducted in accordance with the Cochrane Handbook^14^ and Preferred Reporting Items for Systematic Reviews and Meta-Analyses [PRISMA] recommendations for reporting systematic reviews.^15^

### 3.1. Data sources and search strategy

We designed a comprehensive search strategy and searched PubMed/MEDLINE, Embase, and Scopus up to October 2021 to identify eligible studies. A hand-search of abstracts from the annual meetings of Digestive Disease Week, the American College of Gastroenterology, the European Crohn’s and Colitis Organisation, and the United European Gastroenterology Week, up to 2021, was also performed.

The search query employed both an exhaustive list of keywords and index terminology whenever possible. The following key words and corresponding Medical Subject Heading/Entree terms were used: ‘elastography’, ‘ultrasound elastography’. The Medline search strategy was: [Ultrasound elastography] OR [elastography] OR [shear wave] OR [acoustic radiation force impulse imaging] OR [strain elastography] AND [inflammatory bowel disease] OR [Crohn’s disease] OR [Colitis, Ulcerative’: Mesh] OR [Crohn] AND [histology] OR [histopathological] OR [microscopic] OR [histopathology] OR [pathology] NOT animals. The full search strategy is available in the Supplementary material. No date or language filters were employed in the search. The literature search was performed and verified by two authors [FF, ADB].

### 3.2. Inclusion and exclusion criteria

The inclusion criteria were: a] studies investigating the application of USE in IBD patients; b] studies including a histopathological confirmation of the analysed bowel segment; c] studies on different USE modalities [i.e., SWE and SE]. No restriction on the type of study was applied. Full-text papers, conference abstracts, and case reports were included. Studies on paediatric populations [<16 years old] were excluded. All editorials, letters, or review articles were excluded. Animal studies were excluded as recommended in the Cochrane Handbook of Systematic Reviews of Interventions.^14^

### 3.3. Selection process, data extraction, and quality assessment

Two review authors [FF, ADB] independently screened the titles and abstracts yielded by the search. Full reports were obtained for all titles that appeared to meet the inclusion criteria or where there was any uncertainty. Disagreements were resolved through collegial discussion. The reasons for excluding trials were recorded. When there were multiple articles for a single study, the latest publication was used. The studies were reviewed for patients’ selection and features, technical aspects, USE, and histological assessment. When the USE assessment was done through classes based on the analysis of qualitative colour maps, it was considered semi-quantitative; when the USE measurements were reported as absolute values, it was considered a quantitative assessment. Finally, when the USE measurements were not ordered into classes of severity, it was considered as a qualitative assessment.

The quality of the included studies was assessed with the Quality Assessment of Diagnostic Accuracy Studies [QUADAS-2] checklist.^16^ This tool includes four domains: patient selection, index test, reference standard, and flow and timing. The risk of bias is evaluated across all four domains, and the first three domains are also assessed in terms of concerns regarding applicability. The QUADAS-2 allows expression of an overall judgment as ‘low risk of bias’ or ‘low concern regarding applicability’ in case of assignment of ‘low’ to most/all domains relating to bias or applicability. If a study is judged ‘high’ or ‘unclear’ regarding one or more domains, then it may be judged ‘at risk of bias’ or as having ‘concerns regarding applicability’.

## 4. Results

**Figure 2.**
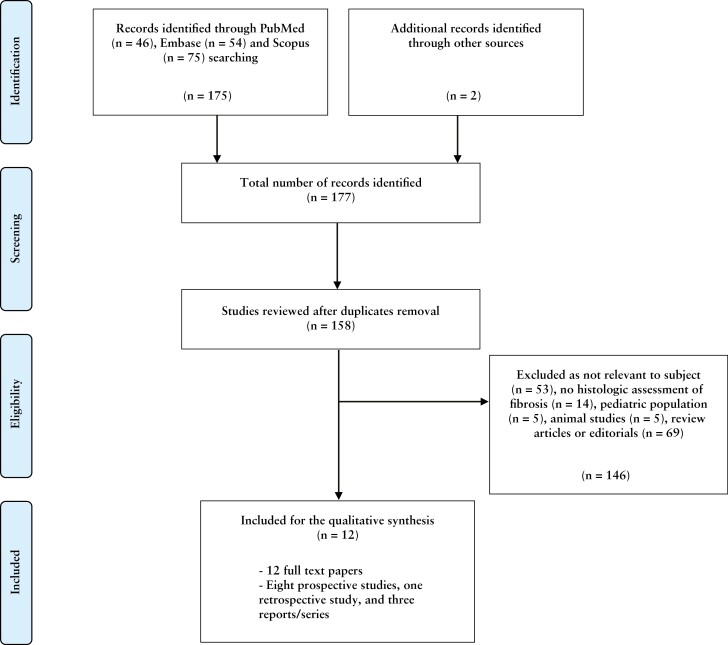
Preferred Reporting Items for Systematic Reviews flow diagram.

The literature search revealed 177 publications: after excluding any duplicates, 158 were screened. After reviewing the title and abstract and, if necessary, the full publication, 146 records were rejected. After the reviewing process, a total of 12 publications met the inclusion criteria,^17^ all full-text [100%]. Figure 2 illustrates the screening and the selection process. All studies were single-centre experiences. The study design of the included papers was as follows: eight prospective studies [66.7%],^18–25^ one retrospective study [8.3%],^26^ and three case reports/series [25%].^17,27,28^ In five studies there was a control group or a comparison with healthy tissue.^17,18,20,21,24^ All papers included were written in English and published between 2011 and 2019. Table 1 presents all the included studies. According to the QUADAS-2 checklist, most studies were found to be at low or moderate risk of bias^17–26^ [Table 2]; two studies were found to be at higher risk of bias.^27,28^ None of the studies was excluded because of quality concerns.

**Table 1.** Studies included in the analysis.

Reference, first author	Year	Study design	*N* ^a^	UC	CD	US elastography technique	US device	Results on accuracy of USE
Stidham RW^17^	2011	Case series	7		7	SE	Zonare Medical Systems [Z-1], Mountain View, CA	Fibrostenotic bowel was stiffer than the normal tissue resection margins in all subjects [*p* = .0009]
Havre RF^18^	2014	Prospective	9		9	SE	HV900 [Hitachi Medical Corporation, Tokyo, Japan]	Mean SR of 3.33 [SD, 5.21] in CD patients with increased tissue fibrosis [score ≥1]
Dillman JR^19^	2014	Prospective	11	3	8	pSWE/2D-SWE	Acuson S3000 [Siemens Medical Solutions USA, Inc., Mountain View, CA]	High fibrosis vs low fibrosis score segments showed significantly greater mean shear wave speed [*p* = 0.049]
Baumgart DC^20^	2015	Prospective	10		10	SE	Hitachi [Lübbecke, Germany]	A higher collagen content was associated with RTE-assessed strain [*p* <0.001] and tensiometry-assessed strain [*p* <0.001]
Fraquelli M^21^	2015	Prospective	23		23	SE	Philips iU22 [Philips Healthcare, Bothell, WA]	Area under the receiver operating characteristic curve: 0.917 [95% CI, 0.788 to 1.000]
Giannetti A^27^	2016	Case report	1		1	SE	Mylab Twice, Esaote, Italy	The SE pattern of reduced elasticity corresponded to histological fibrosis
Lu C^22^	2017	Prospective	105		105	pSWE	Philips Epiq 5,Philips IU-22 [Philips Healthcare,Bothell, WA] or Acuson S3000, [Siemens Medical Solutions, Malvern, PA]	Moderate correlation between SWE and muscular hypertrophy [r = 0.59, *p* = 0.02]
Serra C^23^	2017	Prospective	26		26	SE	iU22 Philips [Philips, Bothell, WA, USA]	No significant correlation was found between mean SR and fibrosis score [ *p*= 0.877].
Chen YJ^24^	2018	Prospective,cross-sectional	35		35	pSWE/2D-SWE	SuperSonic Imagine S.A., Aix-en-Provence, France	Sensitivity of 69.6% and specificity of 91.7% with AUC of 0.822 [95% CI, 0.685 to 0.960] [*p* = 0.002]
Quaia E^25^	2018	Prospective	20		20	SE	iU22 Philips[Philips, Bothell, WA, USA]	Overall accuracy of SE alone of 30% to 35% in distinguishing fibrotic bowel; increased accuracy when combined with CEUS, US
Ding SS^26^	2019	Retrospective	25		25	SE, pSWE/2D-SWE	S2000 [Siemens Medical Solutions, Mountain View, CA]	Sensitivity of 75% and specificity of100%, accuracy of 96% [*p* <.05] for p-SWE [cutoff value >2.73 m/s]
Thimm MA^28^	2019	Case report	3		3	pSWE	EPIC scanner [Philips Healthcare]	Increased level of stiffness [1.58 m/s] consistent with histological fibrosis

IBD, inflammatory bowel disease; UC, ulcerative colitis; CD, Crohn’s disease; US, ultrasound; USE, ultrasound elatography; SE, strain elastography; pSWE, point shear wave elastography; 2D-SWE, two-dimensional shear wave elastography; NS, not specified; SR, strain ratio; SD, standard deviation; AUC, area under the curve; SD, standard deviation; CI, confidence interval; CEUS, contrast enhanced US; RTE, real-time elastography.

Exclusively IBD patients.

**Table 2.** Tabular presentation of QUADAS-2^16^ results of the included studies.

Reference, first author	Risk of bias			Flow and timing	Applicability concerns			Overall risk	Author’s note
	Patient selection	Index test	Reference standard		Patient selection	Index test	Reference standard		
Stidham RW^17^	☺☹	☺	☺	☹	☺	☹	☹	Low/ moderate	Small sample size, intra-individual comparison only, concerns about validation and reproducibility
Havre RF^18^	☺	☺	☺	☹	☺	☹	☺	Low	Small sample, the primary observer was not blinded, *ex vivo* setup
Dillman JR^19^	☺	☺	☹	?	☺	☺	☺	Low	Small sample size, *ex vivo* setup, single operator, concerns about validation and reproducibility
Baumgart DC^20^	☺	☺	☺	☹	☺	☺	☺	Low	Small sample size, inter- and intra-observer variability not assessed
Fraquelli M^21^	☺	☺	☺	☹	☹	☺	☺	Low	Small sample size, ultra-selected patients, concerns about validation and reproducibility
Giannetti A^27^	☹	☹	☺	?	☹	☺	☺	Moderate/ high	Case report, concerns about validation and reproducibility
Lu C^22^	☺	☺	☹	☺	☹	☺	☺	Low	Possible patients’ selection bias, two different US devices, concerns about validation
Serra C^23^	☺	☺	☹	☺	☺	☹	☺	Low	Small sample size, no control group, concerns about validation and reproducibility
Chen YJ^24^	☺	☺	☺	☹	☺	☺	☺	Low	Small sample size, intra-individual comparison only, concerns about validation
Quaia E^25^	☺	☺	☹	☺	☺	☺	☹	Low	Small sample size, concerns about the reference standard used, validation and reproducibility
Ding SS^26^	☺	☺	☹	☹	☺	☺	☹	Moderate	Small sample size, retrospective design, concerns about validation and reproducibility
Thimm MA^28^	☹	☹	☹	?	☹	☺	☺	Moderate/ High	Case report, concerns about validation and reproducibility

The QUADAS tool^16^ consists of four domains that assess: patient selection [was a consecutive or random sample of patients enrolled? was a case-control design avoided? did the study avoid inappropriate exclusions? could the selection of patients have introduced bias?]; index test [were the index test results interpreted without knowledge of the results of the reference standard? if a threshold was used, was it prespecified? could the conduct or interpretation of the index test have introduced bias?]; reference standard [is the reference standard likely to correctly classify the target condition? were the reference standard results interpreted without knowledge of the results of the index test?]; and flow of patients throughout the study design and timing of the index tests and reference standard [was there an appropriate interval between index tests and reference standard? did all patients receive a reference standard? did all patients receive the same reference standard? were all patients included in the analysis?].

US, ultrasound; ☺ low risk; ☹ high risk; ?, unclear.

Overall, a total of 275 IBD patients were included: 272 CD [98.9%] and three UC [1.1%]. Approximately half of the affected patients were male at 138 [50.2%], 121 were female [44%], and in 16 cases the gender was not specified [5.8%]. The age of the included patients ranged from 16 to 70 years.

### 4.1. Ultrasound elastography technique

Seven studies [58.3%] investigated the strain elastography [SE] as elastographic technique,^17,18,20,21,23,25,27^ and shear wave elastography [SWE] was adopted in four studies [41.6%]^19,22,24,28^; in one study only [0.1%], authors explored the use of both techniques [SE, SWE].^26^ The ultrasound elastography techniques and the devices used are presented in Table 1. As concerns the timing and the modality of the ultrasonographic evaluation, in most of the studies [10/12, 83.3%] the elastography assessment was pursued trans-abdominally and pre-operatively,^16,19–27^ and in two studies [16.7%] the assessment was *ex vivo* on the resected intestine.^18,19^ The evaluated segments were small and large bowel in the majority of the papers [66.7%]^18–24,26^ and small bowel only in four studies [33.3%].^17,25,27,28^Table 3 summarises further technical details of the included studies. The USE assessment was mainly semi-quantitative [i.e., with a colour map] in four studies,^17,18,20,25^ purely quantitative and qualitative in five^19,22–24,28^ and one paper,^27^ respectively, and both semi-quantitative and quantitative in the works by Fraquelli *et al*. and by Ding *et al.*^21,26^ In most studies, the USE was performed by a single operator,^17–19,24,26–28^ and in five studies there was more than one sonographer [two to five].^20–23,25^ The inter-observer agreement was estimated as moderate and excellent by Quaia *et al.* and Fraquelli *et al.*, respectively.^21,25^

**Table 3.** Technical details of the included studies.

Reference, first author	Histological specimen	Examined intestine	*Ex vivo*/pre-operatively	Control group/comparison with healthy tissue	USE assessment	Histologic assessment
Stidham RW^17^	Surgical resection	Small bowel	Trans-abdominal, pre-operatively	Yes	Semi-quantitative	Semi-quantitative
Havre RF^18^	Surgical resection	Small- and large-bowel	*Ex vivo*	Yes	Semi-quantitative	Semi-quantitative
Dillman JR^19^	Surgical resection	Small- and large-bowel	*Ex vivo*	No	Quantitative	Semi-quantitative
Baumgart DC^20^	Surgical resection	Small- and large-bowel	Pre-operatively and *ex vivo*	Yes	Semi-quantitative	Semi-quantitative
Fraquelli M^21^	Surgical resection	Small- and large-bowel	Trans-abdominal, pre-operatively	Yes	Semi-quantitative and quantitative	Semi-quantitative
Giannetti A^27^	Surgical resection	Small bowel	Trans-abdominal, pre-operatively	No	Qualitative	Qualitative
Lu C^22^	Surgical resection	Small- and large-bowel	Trans-abdominal, pre-operatively	No	Quantitative	Semi-quantitative
Serra C^23^	Surgical resection	Small- and large-bowel	Trans-abdominal, pre-operatively	No	Quantitative	Semi-quantitative
Chen YJ^24^	Surgical resection	Small- and large-bowel	Trans-abdominal, pre-operatively	Yes	Quantitative	Semi-quantitative
Quaia E^25^	Endoscopic biopsies or surgical resection	Small bowel	Trans-abdominal, pre-operatively	No	Semi-quantitative	Semi-quantitative
Ding SS^26^	Endoscopic biopsies or surgical resection	Small- and large-bowel	Trans-abdominal, pre-operatively	No	Semi-quantitative and quantitative	Semi-quantitative
Thimm MA^28^	Surgical resection	Small bowel	Trans-abdominal, pre-operatively	No	Quantitative	Qualitative

USE, ultrasound elastography.

### 4.2. Histological evaluation

The histological definition of fibrosis was ‘the evidence of increased submucosal collagen deposition’ in all the included studies. The use of Masson trichrome staining was specified in six works.^17,19–22,24^ The histological assessment was semi-quantitative [i.e., based on scores] in all the studies^17–26^ except for two, where the authors evaluated the fibrosis only qualitatively/descriptively.^27,28^ The microscopic evaluation was largely conducted on surgical specimens of resected intestinal tracts,^17–24,27,28^ and Quaia *et al.* and Ding *et al.* included also endoscopic biopsies.^25,26^ Some authors included in the microscopic assessment also the extension of the fibrosis into the tunica muscularis and the muscular hypertrophy^17,22,27^ identified by α-SMA staining, or the loss/reduction of the inflammatory infiltrate.^25^ In greater detail, in the studies by Baumgart *et al.*, Chen *et al.*, Fraquelli *et al.*, Quaia *et al.*, Serra *et al.*, Lu *et al.* and Dillman *et al.,* the thickness of the wall layers was measured [µm], a score of inflammation was adopted, and fibrosis was graded [+/- Masson trichrome or van Gieson staining] in progressive semi-quantitative classes, the last class being characterised by extensive transmural fibrosis.^19–25^ In the studies by Havre *et al.* and Ding *et al.,* a semiquantitative assessment of inflammation and fibrosis on the tissue samples was also used [the progressive classes were not detailed].^18,26^Table 4 summarises the adopted histological scores in the included papers.

**Table 4.** Histological scores adopted in included studies.

Reference, first author	Inflammation score	Fibrosis score	Main achievements
Dillman JR^19^	0 = o inflammation 1 = ow level of inflammation with scattered infiltrating mononuclear cells 2 = oderate inflammation with multiple foci 3 = igh level of inflammation with increased vascular density and marked wall thickening 4 = aximal severity of inflammation with transmural leukocyte infiltration and loss of goblet cells^a^	0 = o architectural distortion, no abnormal Masson trichrome staining 1 = o architectural distortion, mild abnormal Masson trichrome staining in mucosa/submucosa 2 = ubstantial abnormal mucosal/submucosal Masson trichrome staining with modest distortion of architecture but without obscuration of the mucosal/submucosal border 3 = ransmural fibrosis with abnormal Masson trichrome staining in all histological layers, transmural architectural distortion	Statistically significant difference of SWE throughout the classes of fibrosis [low vs high fibrosis AUC max = 0.91]
Baumgart DC^20^	1.presence of intraepithelial neutrophils [0 = none, 1 = few, 2 = excessive] 2.goblet cell reduction in crypts with surrounding neutrophils [0 = none, 1 = little, 2 = excessive] 3.excess of neutrophils in lamina propria [0 = none, 1 = few, 2 = excessive] 4.presence of crypt atrophy [0 = absent, 1 = present] 5.presence of fibrosis [0 = absent, 1 = present]^b^	0 = o increased collagen deposition 1 = ncreased collagen deposition in submucosa 2 = ncreased collagen deposition in submucosa and mucosa 3 = ncreased collagen deposition in muscularis mucosa, submucosa, and mucosa, as well as thickening and disorganiation of the muscularis mucosa 4 = ncreased collagen deposition in muscularis propria, muscularis mucosa, submucosa, and mucosa 5 = increased collagen throughout all layers, including serosa^e^	Higher collagen content in affected versus unaffected segments associated with RTE-assessed strain [*p* <0..001]
Fraquelli M^21^	acute inflammatory score, and chronic inflammatory score ^‡^	Mild/moderate versus severe^c^	Strain ratio was significantly correlated with the bowel fibrosis [AUC for severe fibrosis = 0.917]. Bowel fibrosis was the only independent determinant of the strain ratio at multivariate analysis
Lu C^22^	1.acute inflammatory score [ulceration, cryptitis, crypt abscess, lamina propria neutrophilic infiltration] 2.chronic inflammatory score [lamina propria lymphoplasmacytic cellularity, lamina propria eosinophilic infiltration, crypt architecture alteration]	0 = one 1= <33% 2= >33% and <66% 3= 66%	Correlation observed between SWE and muscular hypertrophy No correlation between SWE and fibrosis score
Serra C^23^	0 = o polymorphonuclear or mononuclear leukocytes infiltrates 1 = Mild] cryptitis, leukocytes infiltrates limited to mucosa 2 = Moderate] cryptitis, crypt abscess, and leukocytes infiltrates until the submucosa 3 = [Severe] transmural inflammation with leukocytes infiltrates in all the layers^c^	0 = one or normal fibrosis 1 = inimal fibrosis limited to submucosa 2 = Submucosaland muscular layer fibrosis <30% 3 = ubmucosal and muscular layer fibrosis between 30% and 60%, with preserved layers 4 = Massive transmural fibrosis >60%, effacement of normal layers^c^	No correlation between SE and fibrosis score
Chen YJ^24^	0 = o inflammation or distortion 1 = amina propria inflammation only 2 = ubmucosal foci of inflammation and/or foci of transmural inflammation 3 = ignificant, dissecting, confluent transmural inflammation	0 = o fibrosis 1 = inimal fibrosis in submucosa or subserosa 2 = ncreased submucosal fibrosis, septa into muscularis propria and/or septa through muscularis propria, increase in sub-serosal collagen 3 = ignificant transmural scar, marked sub-serosal collagen	SWE was significantly different throughout the classes of fibrosis [mild/moderate vs severe fibrosis AUC = 0.822; sensitivity = 69.6%; specificity = 91.7%]. No difference in SWE observed with respect to inflammation classes. Combined SWE + US showed a moderate agreement with the classes of strictures
Quaia E^25^	1.mucosal ulceration [grade 0–3] 2.edema [grade 0–3] 3.quantity [grade 0–3] of neutrophilic infiltration 4.depth [grade 0–4] of neutrophilic infiltration^c^	Sections were scored as positive for fibrosis if at least moderate fibrosis was observed which involved the submucosa or deeper layers^d^	SE was able to differentiate between fibrosis and inflammation with a maximal AUC of 0.885

RTE; real-time elastography; SE, strain elastography; AUC, area under the curve; SWE, shear wave elastography.

According to Likert-like scales.

According to Bataille F, Klebl F, Rümmele P, *et al*. Histopathological parameters as predictors for the course of Crohn’s disease. *Virchows Arch* 2003;**443**:501–7.

According to Chiorean MV, Sandrasegaran K, Saxena R, *et al.* Correlation of CT enteroclysis with surgical pathology in Crohn’s disease. *Am J Gastroenterol* 2007;**102**:2541–50 and/or Borley NR, Mortensen NJ, Jewell DP, *et al.* The relationship between inflammatory and serosal connective tissue changes in ileal Crohn’s disease: evidence for a possible causative link. *J Pathol* 2000;**190**:196–202.

According to Gupta RB, Harpaz N, Itzkowitz S, *et al.* Histologic inflammation is a risk factor for progression to colorectal neoplasia in ulcerative colitis: a cohort study. *Gastroenterology* 2007;**133**:1099–105.

According to Theiss AL, Fuller CR, Simmons JG, Liu B, Sartor RB, Lund PK. Growth hormone reduces the severity of fibrosis associated with chronic intestinal inflammation. *Gastroenterology* 2005;**129**:204–19.

In addition to USE, the histological fibrosis was compared with further ultrasound features such as conventional US and/or contrast enhanced US [CEUS] in seven publications.^21–25,27,28^

### 4.3. Accuracy of elastography in detecting fibrosis

Overall, an accurate differentiation of inflammatory from prevalently fibrotic intestinal tracts, compared with histology, was found in all the included papers,^17,19–22,24–28^ except for the ones by Havre *et al.* and Serra *et al.*^18,23^ These authors concluded in their studies that USE could not accurately distinguish the grade of inflammation from fibrosis.^18,23^ As concerns USE accuracy in detecting fibrosis, point-SWE was found to perform better compared with SE and ARFI by Ding *et al.*^26^ Taking together the assessments of USE in all the included studies, their accuracy varied from 35 to 91%.^17^ In detail, Baumgart *et al.* observed significantly higher mean strain ratios in unaffected compared with affected intestinal tracts [mean +/- standard deviation, 77.1 +/- 21.4 vs 13.3 +/- 11.2, *p* <0.001].^20^ In this study, the affected tracts displayed increased collagen deposition, also significantly associated with USE assessments [*p* <0.001].^20^ Fraquelli *et al.*, in their prospective study on 23 CD patients, reported an extremely accurate discriminatory capability of USE for severe bowel fibrosis (area under the receiver operating characteristic curve [AUROC]: 0.917; 95% confidence interval [CI] 0.788 to 1.000).^21^ In addition, Chen *et al.* graded the fibrosis according to SWE, demonstrating that the mean SWE value of the examined bowel wall was significantly higher in severe fibrosis [23.0 +/- 6.3 KPa] than in moderate [17.4 +/- 3.8 KPa] and mild fibrosis [14.4 +/- 2.1 KPa] [*p* = 0.008].^24^ Finally, the combination of USE with conventional B-mode US and CEUS gained greater accuracy according to most of the authors,^21–25,27,28^ reaching an AUROC of 0.953 [0.887 to 1].^25^

## 5. Discussion

This systematic review illustrates the present understanding of the capability of USE in detecting and quantifying, whenever possible, the degree of fibrosis within the bowel wall of IBD patients.

Collectively, the analysis of the published literature testifies an overall shared moderate-to-good accuracy of USE in detecting histological fibrosis [10/12 studies].^17,19–22,24–28^ In detail, the accuracy of USE varied from 35% to 91% in all the included studies.^17^ However, important concerns are raised regarding the heterogeneity of the USE modalities investigated [SE, SWE], in terms of both the input application/stimulus and the biomarkers analysed [i.e., strain ratio, pSWE, etc.] that do not allow formulation of unequivocal accuracy data. In particular, since SE only allows semi-quantitative assessments of stiffness, these are difficult to compare longitudinally. With respect to technical aspects, all the studies included had a limited cohort of patients [from 1 to 105 patients] and the US devices used were of different manufacturers, the Philips iU22 being the most used^21,23,25^ [Table 1]. These methodological gaps might be only overcome by multicentre studies adopting a common USE equipment.

A further explanation of the variation of USE accuracy observed in our systematic review is the heterogeneity of the bowel segments analysed in the included studies. Indeed, the studies investigating exclusively the ileum in CD patients, where the pathological processes involve the whole bowel wall, reported higher rates of accuracy and better correlation between USE measurements and histology.^17,21,22^

Possible selection bias must be additionally addressed: the investigation of USE in advanced stenosis candidates for surgical resection might have returned higher rates of tissue fibrosis, possibly enhancing the accuracy of USE. The incorporation of a control group and the inclusion of different stages of disease in the study design are necessary to reduce this kind of bias.

The main strength of our systematic review was to address the accuracy of USE in detecting fibrosis exclusively in comparison with histological assessment. To our knowledge, this inclusion criterion was never adopted by previous systematic reviews on the topic of USE in IBD.^29–32^ Of note, the definition of fibrosis was univocally adopted by all the studies, but the histological assessment varied from semi-quantitative^17–26^ to merely qualitative,^27,28^ thus limiting the uniformity of the data and a direct comparison between the studies. A further main finding emerges from our analysis, which is the lack of a standardised histological score to quantify fibrosis. Indeed, a strong discrepancy with respect to the reference standard adopted [i.e., also endoscopic biopsies, which limits the proper assessment of the submucosal fibrotic changes] and the histological quantification of fibrosis was found between the included studies.

It appears clear that USE has been more extensively investigated in CD than in UC [only one study included, three UC patients], but with the growing adoption of US also in the monitoring of UC this trend will reasonably change in the near future.

Interestingly, several publications suggested the integration of USE with conventional B-mode US and CEUS in order to gain greater accuracy.^21–25,27,28^ Indeed, this concept has been broadly explored and is well known by experienced bowel sonographers who are used to combining different qualitative and quantitative features within activity scores.^33,34^

The matter of operator dependency remains for USE, as for all ultrasonographic methods. When estimated, there was a moderate-to-high inter-reader agreement in SE measurements,^21,25^ and we can speculate that inter-reader agreement might be superior in the case of SWE. A consensus on specific skills and training for USE operators is yet to be specifically established.

Another relevant issue regards the quality assessment of the included studies [Table 2].^16^ Indeed, despite addressing the main methodological features of scientific studies, many limitations could not be addressed [i.e., the exiguous sample size, the lack of validation, and reproducibility] [Table 2].

The main limitation of this work is that no meta-analysis was performed due to the lack of standardisation between the results of the included studies; further limitations derive from the discussed heterogeneity of the included studies [different techniques of USE, different reference standards].

In our view, the so far gathered data on USE deserve endorsement to be incorporated into the management algorithms of IBD, whereas USE does not appear to add any specific information to guide clinical decisions. Indeed, current European Federation of Societies for Ultrasound in Medicine and Biologys guuidelines instruct on USE with a relatively low level of evidence and suggest using it to characterise bowel wall lesions exclusively in CD.^35^

The appeal of USE lies in its non-invasiveness and repeatability. Indeed, in the treat-to-target era a new physiological surrogate endpoint, such as the quantification of the intestinal fibrosis, would be warmly welcome by dedicated physicians.

Our systematic review endorses that elastography cannot replace the tissue specimen yet, at least in the field of bowel ultrasound and IBD. The applicability of this technique to the bowel wall, compared with parenchymal organs might be limited and challenged by the unique features of the intestine [i.e., peristalsis, the peritoneum, the structure in layers].

In conclusion, despite the data gathered so far, the role of USE in the detection and quantification of fibrosis in IBD patients requires additional research with properly designed randomised clinical trials. Moreover, long-term data on patients followed up with USE longitudinally over time are warranted as well.

The data underlying this article are available in its online Supplementary material and upon request to the corresponding author.

## Supplementary Material

jjac082_suppl_Supplementary_Material

jjac082_suppl_Supplementary_Data
