# Supplementary material for: Ultrasound Elastography in Inflammatory Bowel Diseases: A Systematic Review of Accuracy Compared with Histopathological Assessment
Source: J Crohns Colitis. 2022 Jun 13;16(10):1637–46. doi: 10.1093/ecco-jcc/jjac082 (PMC9624288; doi:10.1093/ecco-jcc/jjac082)
Supplement: jjac082_suppl_Supplementary_Material [file jjac082_suppl_supplementary_material.docx]

**Supplementary material: search strategy**

**1) PubMed/Medline**

(((((Ultrasound elastography) OR elastography) OR shear wave) OR acoustic radiation force impulse imaging) OR strain elastography) AND ((((inflammatory bowel disease) OR Crohn's disease) OR "Colitis, Ulcerative"[Mesh]) OR Crohn) AND (((((histology) OR histopathological) OR microscopic) OR histopathology) OR pathology) NOT animals

**2) Embase**

- ('ultrasound elastography'/exp OR ' elastography ') AND ('inflammatory bowel disease'/exp OR 'inflammatory bowel disease') AND ('histology'/exp OR 'histopathology')

**3) Scopus**

- ('ultrasound elastography'/exp OR ' elastography ') AND ('inflammatory bowel disease'/exp OR 'inflammatory bowel disease')
